# “We make choices we think are going to save us”: Debate and stance identification for online breast cancer CAM discussions

**DOI:** 10.1145/3041021.3055134

**Published:** 2017-04

**Authors:** Shaodian Zhang, Lin Qiu, Frank Chen, Weinan Zhang, Yong Yu, Noémie Elhadad

**Affiliations:** † Department of Biomedical Informatics, Columbia University, New York, NY, US; ‡ Apex Data and Knowledge Management Lab, Shanghai Jiao Tong University, Shanghai, China; ♮ Mailman School of Public Health, Columbia University, New York, NY, US

**Keywords:** Complementary and Alternative Medicine (CAM), Online Health Community, Debate Identification

## Abstract

Patients discuss complementary and alternative medicine (CAM) in online health communities. Sometimes, patients’ conflicting opinions toward CAM-related issues trigger debates in the community. The objectives of this paper are to identify such debates, identify controversial CAM therapies in a popular online breast cancer community, as well as patients’ stances towards them. To scale our analysis, we trained a set of classifiers. We first constructed a supervised classifier based on a long short-term memory neural network (LSTM) stacked over a convolutional neural network (CNN) to detect automatically CAM-related debates from a popular breast cancer forum. Members’ stances in these debates were also identified by a CNN-based classifier. Finally, posts automatically flagged as debates by the classifier were analyzed to explore which specific CAM therapies trigger debates more often than others. Our methods are able to detect CAM debates with F score of 77%, and identify stances with F score of 70%. The debate classifier identified about 1/6 of all CAM-related posts as debate. About 60% of CAM-related debate posts represent the supportive stance toward CAM usage. Qualitative analysis shows that some specific therapies, such as Gerson therapy and usage of laetrile, trigger debates frequently among members of the breast cancer community. This study demonstrates that neural networks can effectively locate debates on usage and effectiveness of controversial CAM therapies, and can help make sense of patients’ opinions on such issues under dispute. As to CAM for breast cancer, perceptions of their effectiveness vary among patients. Many of the specific therapies trigger debates frequently and are worth more exploration in future work.

## 1. INTRODUCTION

Complementary and alternative medicine (CAM) is increasingly used by populations worldwide in concert with conventional, evidence-based medicine, particularly for treating and managing chronic diseases and life-threatening illnesses [4, 18, 40, 27]. Yet, motivations and perceptions of CAM adoption have been diverse. For example, it is reported that a majority of alternative medicine users appear to be doing so, not so much as a result of being dissatisfied with conventional medicine, but largely because they find these healthcare alternatives to be more congruent with their own values, beliefs, and philosophical orientations toward health and life [3]. Others have found patients are critical of and skeptical about the efficacy of modern medicine and believe that treatment should concentrate on the whole person and greater knowledge of the physiology of the body [13]. As such, patients may take CAM following personal beliefs, sometimes without informing their care providers [12].

For healthcare practitioners and researchers, it is critical to gain a deeper insight into how CAM therapies are perceived and used by patients. Recent research has also focused on attitudes of physicians and patients toward CAM relying on different study instruments, many of which found incongruent views on effectiveness [12, 24, 2, 28]. Most of these studies are based on rigorous study designs on sampled populations, in which subjects are asked to respond to survey instruments or participate in focus groups.

With the rise of health social media, there is an unprecedented opportunity for researchers to study at scale patients’ behaviors and their attitudes toward daily health-related activities. Specifically, information extraction and text mining methods for health purposes have been applied to content from online health forums [39, 41, 43, 42], Twitter [32, 31, 16], Facebook [5] to identify salient information for and characterize health-related behaviors of individuals. As for CAM, researchers have proposed methods to evaluate quality of content [9] and impact of social media [30], but the vast amount of CAM discussions that occur in online health communities have remained unexplored. In fact, some of the popular communities, such as the breast cancer discussion boards from breastcancer.org, have established subforums dedicated to alternative medicine discussions specifically.

Because CAM usage is linked to personal beliefs and because most of CAMs are not adopted by the medical establishment, one research question for this work is to which extent peer-to-peer CAM-related discussions contain conflicting opinions about CAM adoption and/or efficacy. A secondary set of questions pertain to identifying which specific CAM therapies are more likely to trigger debate amongst patients, and what are the stances of patients overall toward these controversial CAMs.

Our overall objectives are therefore (i) to detect instances of debates about CAM in a community; (ii) to classify patients’ stances toward these therapies; and (iii) to identify which specific CAM therapies are more likely to trigger debates in the community. Our study is carried out in an automated and quantitative fashion, and aims to complement perspectives obtained through qualitative methods.

Critical to our objectives is a set of tools that can locate CAM-related debates in different posts of a community, and can identify the stances of the different debate participants toward the CAM under discussion. In the general domain, natural language processing and information extraction techniques have been leveraged in identifying debates and stances from online discussions [38, 37, 35, 34, 29, 25, 7, 15, 14], but to our best knowledge no study has focused on identifying health-related, especially CAM-related, debates specifically.

One challenge behind debate identification is that debate usually happens in consecutive posts and highly depends on discourse of discussion. This requires the model to capture not only document(post)-level content-based features, but also document-to-document connections. In this study, we rely on a long short-term memory neural network (LSTM) [17] stacked over a convolutional neural network (CNN) [23] to identify CAM-related debates from posts in discussion threads, which captures both features from post content and contextual transitions among adjacent posts. We then rely on a typical CNN to classify participants’ stances towards the issues under debate. We extend our analysis on these debate posts through a qualitative analysis to characterize which alternative treatments trigger debates more frequently than others.

## 2. METHODS

We describe our study pipeline in this section, ranging from data collection, data annotation, debate/stance classification experiments, to exploration of therapy prevalence over the debate posts.

### 2.1 Dataset

We rely on the publicly available breastcancer.org discussion board, one of the most active and popular online breast cancer community. In particular, the forum maintains a variety of sub-forums all related to breast cancer, including a sub-forum for alternative medicine discussions. The entire content of the discussion board was collected in January 2015 [19, 10, 41]. The discussion board is organized in distinct forums, each with threads and posts. In total, 3,283,016 posts organized into 121,474 threads were extracted. In this particular study, we focused on the subforum for alternative medicine, which consisted of 25,013 posts part of 396 discussion threads.

The following pre-processing steps were carried out for the target dataset. For each post, meta-information about the forum and the thread in which it was published was kept, along with author ID and creation date. Content of each post was then pre-processed by (i) removing all non-textual content (e.g., substituting emoticon icons with emoticon-related codes); and (ii) identifying sentence boundaries using the open-source tool OpenNLP [1].

### 2.2 Annotation

To assemble a gold standard of posts with debate information, we relied on a manual annotation process. Two annotators (SZ and FC) coded each post according to two binary labels: whether the post is involved in a debate and whether the opinion conveyed in the post in general is for or against alternative medicine usage. The annotation process started with a pilot annotation of 50 posts, in which the annotators made sense of the task by deciding which types of debates of interest to identify. The pilot annotation led to a consensus on three types of debates to be considered: CAM debate (debates over effectiveness/impact/side effects of CAM), BC debate (debates over other cancer-related topics), and other debates amongst members. The two annotators then annotated 100 posts each to calculate inter-rater agreement. After resolving disagreements on the double-annotated 100 posts, the remaining part of dataset is evenly split and coded by single annotator only.

The first annotation task, deciding whether a post is involved in a debate, is heavily dependent on the context: how the author interacts in this post with other members in the thread, and what the general theme of the thread is. As such, to construct our gold standard, we sampled posts from entire threads rather than individual posts throughout the community. For sampled threads with a reasonable number of posts, the annotators annotated all posts in the thread. However, for some giant threads, which often occur in such communities, the annotators annotated the first 300 posts in the thread. Overall, 1,966 posts within 73 threads were annotated. As previously mentioned, we are interested in controversial topics which trigger debates involving opposing opinions, rather than treatment options that are comprehensively accepted and mostly uncontested. As such, a “debate” in our definition must involve different stances from different participants, and should have some degree of opposing interactions. In other words, a post simply stating an opinion but not disagreeing explicitly or implicitly with another’s opinion, as well as receiving no opposing responses from other persons, would not be considered as a debate post, even if it represents a stance on the issue.

For the second task, stance identification, only posts identified in the previous step as CAM-related debates were considered. Specifically, a “con-CAM” stance was annotated, when the post’s author opposes the usage of the specific CAM under discussion, are suspicious of its effectiveness, or concerned about its side effect. Any other opinion, including willingness to try a CAM, defending its effectiveness, or describing the outcome objectively, was considered as a “pro-CAM” stance.

### 2.3 Automated Debate/Stance Detection

#### 2.3.1 Neural networks

**Figure 1:**
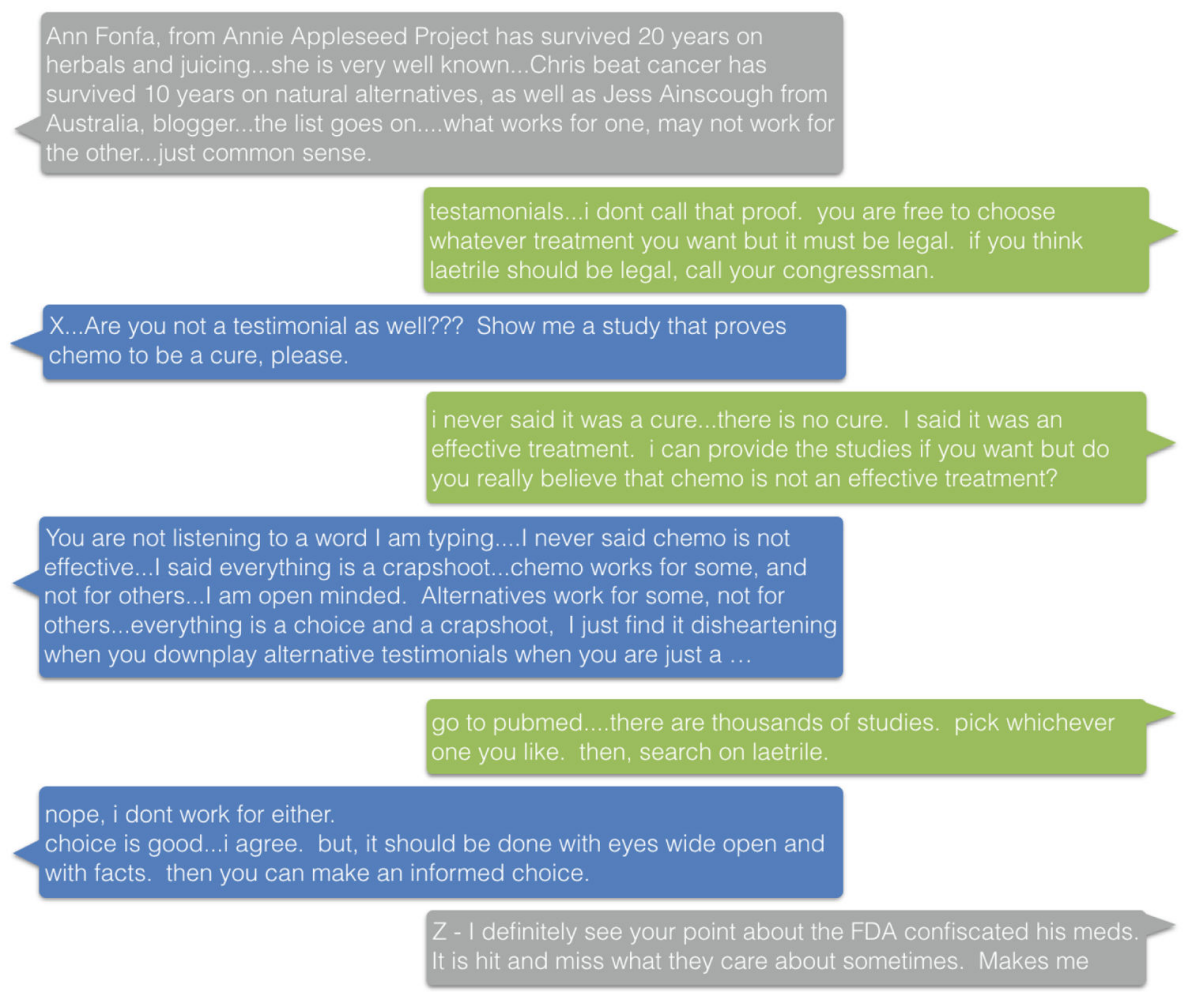
An example debate in thread as the input of our model. Green and blue posts were published by two users engaged in the debate with opposing opinions respectively. Grey posts are not engaged in the debate, but provide as context. User names are removed from the text and replaced by X, Y, and Z, from which it could be seen that debate detection is highly context-dependent.

**Figure 2:**
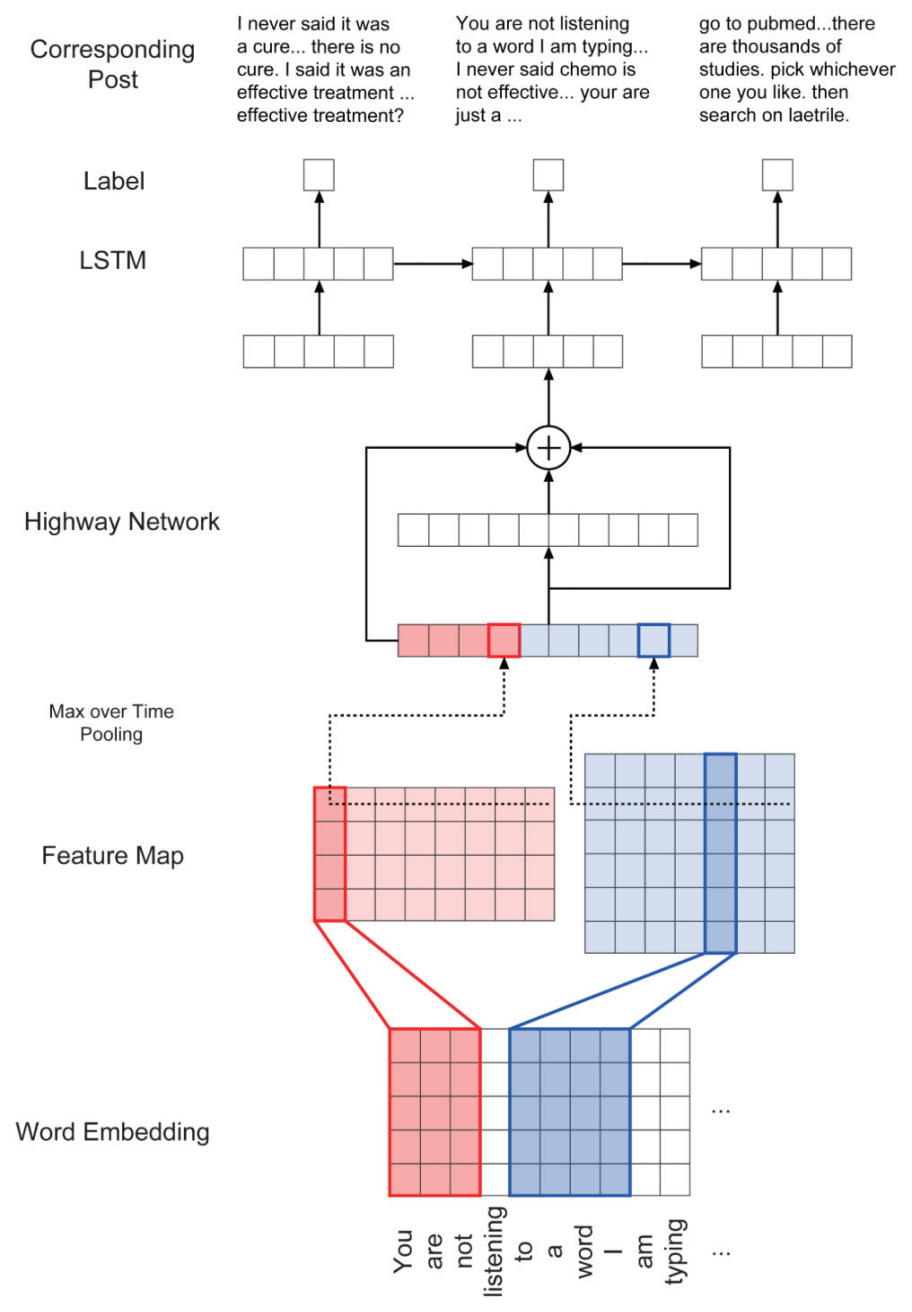
The architecture of our model for debate detection, motivated by [21].

To automate debate and stance detection from the breast cancer forum, we built two neural networks to handle the two tasks, respectively. The first task, debate detection, can be formulated as a document classification task within a sequence of consecutive posts in threads. Adjacent posts in threads are usually highly related with respect to topic, sentiment, and whether they are involved in the same debate. Figure 1 shows an example of a series of debate posts in a thread with context. To build a classifier that can capture such post-to-post connections in context, motivated by the network structure in [21], we adopt a neural network architecture as shown in Figure 2. The original neural network used a CNN to capture character-level features of words, followed by feeding the output of the CNN to an LSTM to model word sequences. We adopt the same architecture in this paper, however, to first capture in-document features of posts by a CNN. This part is also identical to the document classifier created in [20], where word vectors are concatenated and filtered by a convolutional layer. The output of the CNN, where each document is represented as a document vector after the max-pooling layer, is fed into an LSTM so that thematic relations between context posts can be captured. The final output of the LSTM is an indicator whether a post is involved in debate or not, and which type of debate it belongs to.

To describe our model from end to end, we start with the notations. Let 𝒱 be the vocabulary of words, *d* be the dimensionality of word embeddings, and **E** ∈ ℝ*^d×|^*^𝒱^*^|^* be the matrix of word embeddings. Let ℘ be the collection of posts, suppose that a post *p* ∈ ℘ is made up of a sequence of words [*w*_1_*,* …*,w_l_*] where *l* is the length of the post *p*. Then the word-level representation of the post *p* is given by the matrix **W***^p^* ∈ ℝ*^d×l^* where the *j*-th column corresponds to the word embedding of *w_j_*. Then we introduce a CNN to obtain a feature map with the convolutional layer:

(1) $$f^{p}[i]=\tanh(\langle W^{p}[*,i:i+k-1],H\rangle +b),$$

where **H** ∈ ℝ*^d×k^* is a filter (or kernel) of width *k*, **W***^p^*[*, i : i+*k*−1] denotes the *i*-th column to (*i*+*k*−1)-th column of **W***^p^* and 〈**A***,***B**〉 = tr(**AB***^T^* ) is the Frobenius inner product. We also add a bias *b* and apply a widely used nonlinear activation function tanh(*x*) = (*e^x^* − *e*^−^*^x^*)*/*(*e^x^* + *e*^−^*^x^*) here.

After the convolutional layer, we take the *max-over-time* pooling:

(2) $$l^{p}=\max_{i}f^{p}[i]$$

to select the salient word patterns in the post as the feature corresponding to the filter **H**. We do this process with a total of *h* = 400 filters **H**_1_*,* …*,***H***_h_* with different width *k* (100 filters for *k* = 2*,* 3*,* 4*,* 5 respectively), then $l^{p}=[l_{1}^{p},\ldots ,l_{h}^{p}]$ is the extracted feature representation of the post *p*.

We could directly use **l***^p^* as the input at each time in the LSTM model. Instead, we apply a *highway network* to obtain a new set of features:

(3) $$z=t⊙g(A_{H}l+b_{H})+(1-t)⊙l,$$

where ⊙ is the element-wise multiplication operator, *g* is a nonlinear activation function, **t** = *σ*(**A***_T_* **l** + **b***_T_*) is called the *transform* gate, indicating the part of information to perform non-linear transformation, and the term (**1** − **t**) is called the *carry* gate, indicating the part of information to reserve.

Finally, the processed representation **z***^p^* of the post *p* is fed into the LSTM [17]. LSTMs solve the problem of learning distant dependencies by introducing a memory cell vector **c***_t_* at each time step. Specifically, each step of an LSTM takes **x***_t_,* **h***_t_*_−1_*,* **c***_t_*_−1_ as input and produces **h***_t_,* **c***_t_* as output via the following equations:

(4) $$\begin{array}{l} i_{t}=\sigma (U^{i}x_{t}+V^{i}h_{t-1}+b^{i})\\ f_{t}=\sigma (U^{f}x_{t}+V^{f}h_{t-1}+b^{f})\\ o_{t}=\sigma (U^{o}x_{t}+V^{o}h_{t-1}+b^{o})\\ g_{t}=\tanh(U^{g}x_{t}+V^{g}h_{t-1}+b^{g})\\ c_{t}=f_{t}⊙c_{t-1}+i_{t}⊙g_{t}\\ h_{t}=o_{t}⊙\tanh(c_{t}) \end{array}$$

Here *σ*(·) and tanh(·) are the element-wise sigmoid and hyperbolic tangent functions. **i***_t_,* **f***_t_,* **o***_t_* are input, forget and output gates. **x***_t_* is the proceeded post representation **z** we described above. At the first time step *t* = 1, **h**_0_ and **c**_0_ are initialized to zero vectors. Parameters in the LSTM are **U***^j^,***V***^j^,* **b***^j^* for *j* ∈ {*i, f, o, g*}.

Consider a post sequence *p*_1:_*_t_* = {*p*_1_*,* …*, p_t_*}, we formulate whether a post is involved in a debate with a 2-class classification problem and determine which type of debate it belongs to with a 4-class classification problem. The label sequence of *p*_1:_*_t_* is denoted as *y*_1:_*_t_* = {*y*_1_*,* …*, y_n_*}. We train two models to solve these two problems with the manual labels as we describe in Section 2.2 by minimizing the negative log-likelihood (NLL) of the post sequence:

(5) $$L=-\sum_{t=1}^{T}\log\mathrm{Pr}(y_{t}|p_{1:t-1})$$

Here *T* is the number of posts from a thread. We formulate the probability term with a softmax:

(6) $$Pr(y_{t}=j|p_{1:t-1})=\frac{\exp(h_{t}z^{j}+b^{j})}{\sum_{j^{\prime }\in J}\exp(h_{t}z^{j^{\prime }})+b^{j^{\prime }}}$$

Where 𝒥 is the set of labels, **z***^j^* ∈ ℝ*^d^* is the parameter vector corresponding to the label *j*, *b^j^* is a bias term. We optimize the NLL function by back-propagation.

For stance identification, we simply use a CNN network for post classification created in [20]. Output of the classifier is whether the member is supporting or opposing CAM usage if the post is indeed in a CAM-related debate.

In both classifiers, we used the word2vec tool’s CBOW model to initialize the word vectors [26], and set vector size *N* = 50, iteration number 100 and all other parameters default.

#### 2.3.2 Logistic regression

To show the effectiveness of our neural network method, we also built logistic regression classifiers with lasso regularizations [36] for debate detection and stance identification respectively, and compare the performance with their neural network-based counterparts described above. Three types of features were leveraged for the logistic regression model: thread level features, post level features, and lexical features. Descriptions of features are given in Table 1.

Most of thread- and post-level features were based on meta-information of posts and post authors, as well as shallow information extraction or keyword matching from the content. For NumNeg and NumPos, we simply looked up two adjective lists: glad, happy, relieved, grateful, excited, thrilled, thankful, great, lucky, pleased, blessed, fortunate, hopeful, inspiring, encouraging; and scared, sad, anxious, embarrassing, disappointing, confused, heartbreaking, frightened, frustrated, angry, upset, distress, stress, discouraging, as well as their morphological variants (e.g. frustrated → frustrating). NumCAM feature was generated based on following manually curated list of keywords which appear frequently in the forum: alternative, CAM, ginseng, marijuana, supplement, cream, massage, TCM, gerson, laetrile. For NumOverlap, stopwords were excluded. NDisagree also included negated “agree”s such as “don’t agree”.

For topic modeling, we relied on the Latent Dirichlet Allocation (LDA) model [6] to generate word clusters and to calculate a topic distribution for each post. The LDA topics were trained over the entire unannotated corpus extracted from the breast cancer forum described in previous section. We set the hyper-parameters of LDA model experimentally as follows: *α* = 0.5, *β* = 0.05 and *k* = 15. The W2V features were simply obtained through training word2vec, as described previously in how we initialize the word vectors for the CNNs.

#### 2.3.3 Experimental setup

For debate classification, two separate sets of experiments were carried out. The first set of experiments considered a binary choice between debate and non-debate without taking debate type into account. This task formulation aims at examining in general how difficult it is to identify debates automatically in such an online health community. The second set of experiments took type of debates into account, casting the task as a 4-class categorization of CAM debate, BC-related debate, other debate, and non-debate.

The debate classifier and the stance classifier were cross-validated (5 folds) on the annotated dataset and the subset of posts annotated as in CAM debate, respectively. Classification performance was evaluated using precision, recall, and F scores. The debate classifier was then applied to the entire alternative medicine sub-forum to identify unknown debates automatically, followed by stance prediction by the stance classifier for those posts identified as being in CAM debates.

### 2.4 Coding for specific CAM therapies in debate posts

We carried out a manual analysis to identify which specific CAM therapies are under dispute in the community. We randomly sampled 500 posts from the 3,166 CAM-related debate posts (from 116 threads), as identified in the previous step by our classifier from the entire unannotated dataset of CAM forum. To ensure that each thread is represented in the sampled set and to get around over-sampling posts from massively long threads, we made sure that at least one post from each thread was sampled, in accordance with the length of the different threads. This resulted in a total of 523 sampled posts.

Two annotators (SZ and NE) coded the sampled posts as (i) not debate (i.e., the classifier mis-categorized the sampled post as debate); (ii) not CAM-related (e.g., posts with a debate, but about rules of conduct in the community, or any topic not directly related to CAM); (iii) general CAM debate (e.g., debate post about choosing CAMs as an alternative to chemotherapy); or (iv) specific CAM therapies or groups of therapies (e.g., nutritional supplements). Because some specific therapies had a very high number of threads discussing them, they were assigned their own code (e.g., Gerson diet was kept a separate code from the more general diet code).

The stance classification was also applied to the sampled posts. At the end of this process, we thus can assess in our sample of posts (i) what CAM therapies are prevalently under debate; and (ii) the participant’ stances towards these treatments.

## 3. RESULTS

### 3.1 Data annotation

The two annotators reached an inter-rater agreement measured by Cohen’s Kappa 0.68 on the 100 double-annotated posts with respect to debate identification [22]. Out of the 1,966 annotated posts, 312 were coded as debates after resolving disagreements. Specifically, 181 were coded as debates about CAM, 74 as debates about other breast cancer related topics, and 57 coded as general conflicts. Table 2 gives examples of debate discourse out of context for the three types of debates, respectively.

The inter-rater agreement of stance identification between the annotators was 0.77. After resolving disagreements, 181 posts were annotated as in CAM related debates, 123 were annotated as supporting and 58 against CAM usage.

### 3.2 Debate identification

Table 3 lists the precision, recall, and F measures of different methods for the binary classification of a post into debate vs. non-debate. The baseline system always classifies a post as debate.

For each experiment, we calculated 95% confidence intervals by re-sampling the 5 folds for 5 times from the dataset and assuming that the performance scores are normally distributed. As such, we have 25 sample sets for each experiment to calculate the confidence interval, which can be used to measure whether differences amongst systems are indeed significant. It can be seen that our LSTM+CNN model outperforms logistic regression with statistically significant difference in F score.

Another set of classifiers, which were trained with 4 types of annotated debates (including non-debate), were also evaluated. Table 4 shows detailed performance for each class by using the system based on LSTM+CNN. Since decomposing binary into 4-class makes the dataset sparser and the task more challenging, it is reasonable that accuracies of prediction drop for all categories compared with the binary result. However, the LSTM+CNN model still outperforms logistic regression in identifying non-debate (75.1 vs. 73.9), breast cancer related debate (41.9 vs. 33.8), and other debate (57.2 vs. 56.3).

The 4-class classifier based on LSTM+CNN was then applied to all the 25,013 posts in the alternative medicine sub-forum of breast cancer discussion boards. 5,714 posts in 187 threads were identified as in debate, in which 3,166 posts in 116 threads were CAM, 1,144 posts in 78 threads as breast cancer related, and 1,404 posts in 81 threads as others.

### 3.3 Stance classification

Table 5 shows the performance of stance classification (pro v.s. con) on the gold-standard CAM-related debate posts. Like for the previous experiments, datasets were re-sampled and different models were cross validated. Evaluation is reported through precision, recall, and F score for the con-CAM class. The baseline system simply classified everything as con-CAM. In this task, CNN and logistic regression show no significant difference in performance while CNN is not restricted by any domain knowledge in devising the features and models. The stance classifier based on CNN was then applied to the 3,166 posts identified in previous step as CAM-related debates. 950 of them were identified as opposing CAM usage, which means that around 2/3 posts in CAM related debates are in supportive stances.

### 3.4 Manual Analysis for specific CAM therapies in debate posts

**Figure 3:**
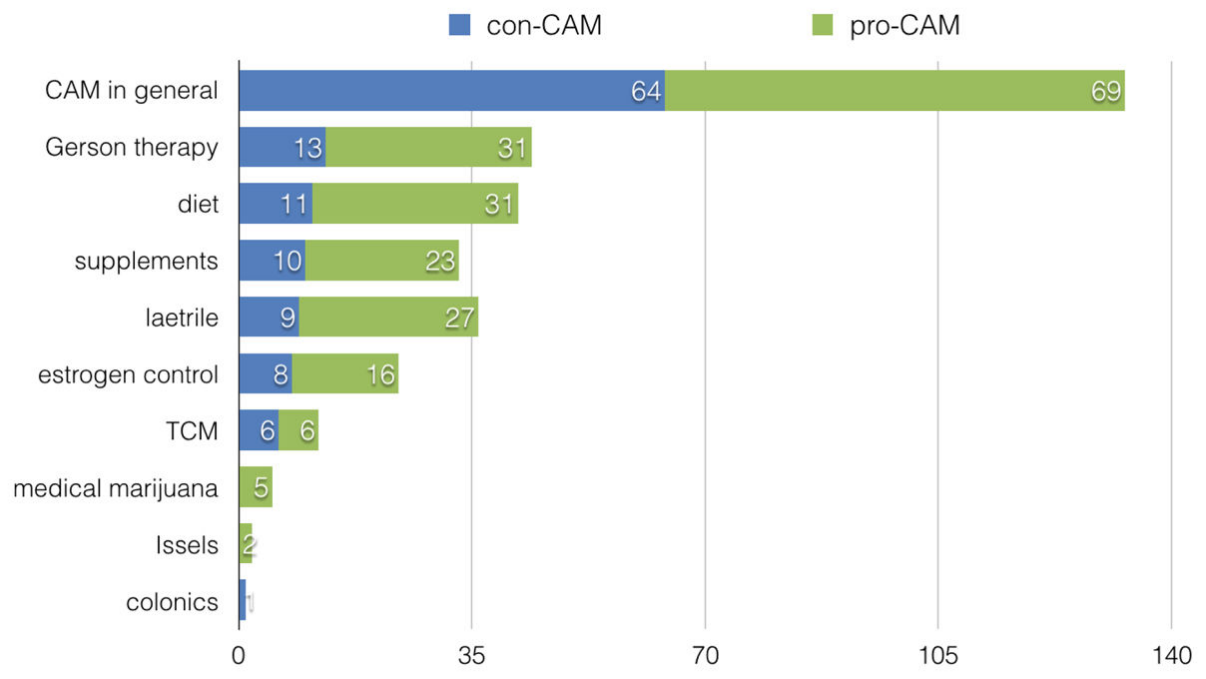
Stances of posts on CAM usage clustered by topics. X axis represents the numbers of posts in pro-CAM and con-CAM stances, respectively.

Out of the 523 sampled posts for manual analysis, 118 were coded as non-debate ones (i.e. classification errors), and 78 were coded as debate but not CAM-related (46 about cancer cause, 16 about cancer diagnosis, and 16 trolling or rules of conduct in the community). The breakdown of the remaining 327 posts coding is provided in Table 6. In addition to the different therapies and their prevalence in the sample posts, Figure 3 illustrates the prevalence of pro-CAM and con-CAM posts for each group.

A large proportion of debates are amongst proponents of CAM therapy and their opponents, on issues such as effectiveness of CAM as a general alternative to conventional treatments like chemotherapy, as well as in addition to conventional treatments. Although all posts in this analysis were from the alternative medicine sub-forum, which is presented to the breast cancer community as a safe place to discuss alternative medicines, there were still a significant number of con-CAM posts present in the sample. Many of the specific alternative treatments, such as Gerson therapy and laetrile, also attract a large amount of debates in the forum, mostly about the scientific validity of the therapies.

## 4. DISCUSSION

### 4.1 Principle Findings

In this study, we proposed an automated methods based on deep neural networks to identify debates and stances of users from online breast cancer forum posts. Experimental results demonstrate that our methods, with very little manual intervention, are effective in quickly locating controversial CAM discussions and identifying user stances. The methods could help make sense of opinions on such health related issues under dispute conveniently. Models used in this paper were domain and community independent, making the approaches portable to any online health communities in which posting in threads is the main way of interaction.

Our experimental results suggest that the LSTM+CNN model is able to capture both in-post and cross-post information, hence outperforms the logistic regression baseline in the task of debate detection. As for stance identification, the CNN model performs roughly on par with logistic regression classifier equipped with rich semantic features. Since size of the dataset is relatively small in our experiment, it could be expected that the neural networks have the potential to amplify the difference between its performance on this specific task and that of traditional machine learning models with manual feature engineering, when the training data set gets larger. By breaking down the effectiveness of features for logistic regression, we found that shallow features such as counting certain keywords and meta-information of posts/threads are effective in identifying debates in discourse and in classifying stances of users. But obtaining such features highly depends on domain knowledge. Distributional semantic representations of texts, such as topic modeling and word embedding, are also helpful, although their importance differs in the two tasks.

To get an evidence of why LSTM+CNN works better for debate detection, we also looked at weights assigned by logistic regression model after training, and found that W2V-sim, NumName, NumOverlap were the top three features associated, either positively or negatively, with debate identification, while NAgree, NumPos, and NumCAM were most correlated with stances. This shows that debate detection is highly context dependent, strongly relying on how authors of posts interact with each other, which justifies the importance of LSTM in connecting the inference of consecutive posts. On the contrary, stance identification is more context independent but domain-knowledge dependent, relying more on domain specific keyword features that are easier to be enumerated manually.

Error analysis shows that many of the false negative errors made by the debate classifier are posts that engage in debates by peacefully stating opinions and providing scientific evidence. One of the most prevalent types of errors made by the stance classifier pertains to those posts with mixed stances, which express both willingness and concern towards CAM usage. This type of posts was coded as pro-CAM in our manual annotation, but can be challenging for the classifier to identify.

### 4.2 How prevalent are debates in CAM discussions compared with other topics?

An interesting question worth exploring is whether CAM as a controversial topic is more likely to trigger debates than other cancer-related issues. To investigate, we applied our best debate classifier, the one based on LSTM+CNN, to the entire breast cancer forum which consists of more than 3 million posts. Results indicate that more than 500,000 (563,231/3,283,016, 17.2%) posts were identified as debate. Compared with the ratio in CAM sub-forum (5,414/25,013, 22.8%), lower proportion of debate posts were found in other sub-forums. However, since our classifier is trained completely on data from the alternative medicine sub-forum, it may import the model bias to underestimate or overestimate the ratio of debate in the other forums.

### 4.3 Characteristics of CAM debates

The most prevalent type of debates is about effectiveness, scientific validity, and usage of alternative therapies in general. Many of such posts are published in threads initiated by newly diagnosed patients or patients suffering from side effects of conventional treatments, who are looking for evidence that supports CAM usage. Debates escalate particularly quickly in discussions when someone considers completely replacing conventional medicine with CAM, focusing on the “alternative” rather than “complementary” part of CAM. Members may be in an opposing stance on such opinions, although many of them in this sub-forum are supposed to be users and hence supporters of CAM. This is consistent with a previous research finding that members of online health communities are able to self-correct misleading opinions [11]. Similarly, debates can be triggered frequently when CAMs are perceived by some members as a standalone treatment of cancer, instead of common perception of CAM as complimentary ways of relieving side effects brought by conventional therapies, such as pain, fatigue, and hot flashes, and to help improve quality of life. Although previous research suggested that CAM use can no longer be regarded as an “alternative” or unusual approach to managing breast cancer given its increasing popularity [8], our study suggests that many patients, even adopting alternative therapies themselves, are still rather cautious about CAM usage. In general, our finding is also consistent with previous survey results that patients not necessarily have positive attitude towards CAM, and that they may not be more optimistic about CAM than health professionals [12].

Interestingly, a small group of firm anti-CAM users, which are sometimes treated by other users as trolls, were also identified by our methods. Sometimes CAM supporters respond to these anti-CAM users in a quite drastic way, such as in following post: “I will never understand why women who do not have breast cancer feel the need to post on a breast cancer board. Why? Consider yourselves lucky….you don’t have cancer! Go live your life!.”

Overall, an automated method to pinpoint controversial therapies or approaches, along with the different stances of community members can be a valuable tool for public health and health communication practitioners. This is particularly valuable in active communities, such as the one we studied, where there can be large amount of content posted every day.

### 4.4 Limitations

This study has several limitations. First, although automated methods are effective ways to quickly locate information of interest, the actual accuracy is subject to the performance of the methods and complexity of the task [33]. Second, our manual coding is based on a sampled set of posts from all debate posts identified. As such, therapies under debate may be missed in the manual analysis. Third, without the total prevalence (including debate and non-debate) of each therapy in the forum, it cannot be determined if therapies with largest number of debate posts are the most controversial ones. The numbers may be biased by the prevalence of the therapies in population and amongst forum members. Finally, results of this study are based on data only from the breast cancer community. Future research should apply the methods in other communities to further validate the machine learning based approaches.

## 5. CONCLUSIONS

In this study, we propose methods based on LSTM and CNN to automatically identify debates along with stances of users from posts of an online health community. Experimental results demonstrate that such methods, complementing qualitative analysis, can effectively locating controversial CAM debates and help make sense of opinions on health issues under dispute. Particularly, LSTM is able to capture post-to-post connections effectively, which is critical in identifying debates in discourse. The study results, based on a breast cancer forum for alternative medicine, suggest that while CAM is widely used in breast cancer management, perceptions of its effectiveness vary among patients. Many of the specific therapies trigger debates more often than others, especially when they are perceived as a replacement of conventional treatments.

## Figures and Tables

**Table 1:** Features used for the logistic regression model.

Thread-level features	Description
NumPost	Number of posts in the thread
NumUser	Number of authors participating in the thread discussion
AvgLen	Average length of post (by word numbers) in the thread
**Post-level features**	**Description**

NumName	Number of mentions of other authors’ names
NumNeg	Number of negative sentiment words
NumPos	Number of positive sentiment words
NumCAM	Number of CAM related keywords
NumOverlap	Number of words that also occur in previous post
Num?	Number of question marks
Num!	Number of exclamation marks
TimeDif	Time difference between current and previous post in thread
Sig	If the author has a signature profile
NAgree	Number of “agree”s
NDisagree	Number of “disagree”s
**Lexical features**	**Description**

LDA	Topic modeling
LDA-sim	cosine similarity between LDA of current and previous post
W2V	Word embedding
W2V-sim	cosine similarity between W2V of current and previous post

**Table 2:** Example posts annotated as three types of debates (presented here out of their thread context). User names are removed from the text and replaced by “X” and “Y”.

Type of debate	Example post
CAM	“Laetrile is snake oil and potentially dangerous. it is illegal to sell it as a cancer treatment because there is zero evidence to so much as suggest that it has any efficacy.”
Breast cancer related	“X, Y is correct. Please read all parts of your link. It clearly states that dcis can be any size. ”
Other	“X, no offense taken and I usually agree with you on the harmless/lonely bit. However, there were some truly over the top comments made that needed to be addressed, IMHO.”

**Table 3:** System performance for binary debate classification with different methods. The baseline system simply classifies everything as debate.

	Precision	Recall	F
Baseline	16.3	100.0	28.0
Logistic regression	64.6 (*±*0.5)	89.6 (*±*0.7)	75.1 (*±*0.7)
LSTM+CNN	68.1 (*±*0.1)	88.9 (*±*0.5)	77.1(*±*0.4)

**Table 4:** System performance for 4-class debate classification by the proposed LSTM-CNN based system.

	Precision	Recall	F
Non-debate	71.4 (*±*0.7)	79.1 (*±*0.2)	75.1 (*±*0.4)
CAM	58.0 (*±*1.8)	73.9 (*±*1.7)	65.0 (*±*1.7)
Breast cancer related	43.4 (*±*2.4)	41.3 (*±*3.1)	41.9 (*±*2.7)
Other	55.1 (*±*2.7)	59.4 (*±*2.8)	57.2 (*±*2.8)

**Table 5:** System performance for binary stance classification with different methods. Precision, recall, and F are calculated for the con-CAM class. The baseline system classifies everything as con-CAM.

	Precision	Recall	F
Baseline	30.9	100.0	47.2
Logistic regression	69.6 (*±*0.8)	70.6 (*±*0.7)	70.1 (*±*0.7)
CNN	69.1 (*±*0.2)	70.9 (*±*0.5)	70.1 (*±*0.4)

**Table 6:** CAM therapies identified through the manual coding, and number of posts identified for each therapy group in the sampled posts.

Code	Examples	#
CAM	General CAM v.s. conventional discussions; Effectiveness and use of CAM v.s. chemotherapy	135
General	CAM v.s. conventional discussions; Effectiveness and use of CAM v.s. chemotherapy	135
Gerson therapy	Effectiveness and scientific validity of Gerson therapy	44
Diet	Effectiveness and/or practice of diets for cure, prevention, and management of breast cancer therapy (gluten free, low carb, hormone free meal, vegan, Ayurvedic, etc.)	42
Supplements	Any supplement whose purpose is not to control estrogen	33
Laetrile	Laetrile or food/supplement that contains laetrile	27
Estrogen control	Therapies/supplements to control estrogen, including DIM, soy, natural replacements for tamoxifen, bioidentical hormones, etc.	24
TCM	Use and effectiveness of Traditional Chinese Medicine for cancer management	12
Med marijuana	Use of medical marijuana for cancer management	5
Issels	Issels treatment	2
Colonics	Colonics treatments	1
